# Among individuals who die of COVID-19, is the percentage who had diabetes actually higher than in those dying of other viral infections?

**DOI:** 10.1186/s40842-026-00325-0

**Published:** 2026-09-15

**Authors:** Neha V. Reddy, Virginia Pate, Til Stürmer, Rachel Wong, Jeremy Harper, Jane E. Reusch, Kenneth J. Wilkins, Jena Tronieri, John B. Buse, Steven G. Johnson, Carolyn T. Bramante

**Affiliations:** 1 Department of Neurology, Mayo Medical School, Rochester, MN USA; 2 Department of Epidemiology, Gillings School of Global Public Health, University of North Carolina at Chapel Hill, Chapel Hill, NC USA; 3 Department of Biomedical Informatics, Department of Internal Medicine, Stony Brook University Renaissance School of Medicine, Stony Brook, NY USA; 4 Owl Health Works, Indianapolis, IN USA; 5 Division of Endocrinology, University of Colorado Denver Anschutz Medical Campus, Denver, CO USA; 6 National Institute of Diabetes and Digestive and Kidney Disease, Bethesda, MD USA; 7 Department of Psychiatry, University of Pennsylvania Perelman School of Medicine, Philadelphia, PA USA; 8 Division of Endocrinology, Department of Medicine, University of North Carolina School of Medicine Chapel Hill, Chapel Hill, NC USA; 9 Institute for Health Informatics, University of Minnesota Twin Cities, Minneapolis, MN USA; 10 Division of General Internal Medicine, Department of Medicine, University of Minnesota Medical School, Minneapolis, MN USA

## Abstract

**Background:**

Early in the COVID-19 pandemic, mainstream news outlets reported that 30–40% of all coronavirus deaths in the United States occurred among individuals with diabetes. This statistic happens to reflect the approximate prevalence of diabetes in older adults, the age group at highest risk for mortality from COVID-19. To provide greater context to this statistic, we sought to quantify both the proportion of decedents from COVID-19 and from influenza who had diabetes.

**Methods:**

For assessing COVID-19 decedents who had diabetes, we used the National COVID Cohort Collaborative (N3C) data enclave, a nationally-representative, harmonized, and de-identified electronic health record database. For assessing influenza decedents who had diabetes, we used Medicare data. We restricted the N3C sample to > 65 years to align with Medicare eligibility.

**Results:**

Among older adults with inpatient mortality due to COVID-19, 46.6% (95% CI: 46.1–47.0) had diabetes. Among older adults with inpatient mortality from influenza, the crude percent with diabetes was 61.2%. When age-standardized to match the N3C COVID-19 data, the percentage of influenza decedents with diabetes was 63.1% (95% CI: 59.1–67.1).

**Conclusions:**

Among older adults with inpatient mortality from respiratory viruses, a very large proportion had diabetes before infection: 63% of influenza decedents and 47% of COVID-19 decedents. Thus, a high proportion of decedents having diabetes is not new or unique to COVID-19. These findings highlight the value of using available data to contextualize health communication to the public.

## Background

In 2022, several mainstream news outlets headlined that 30 to 40% of individuals dying from COVID-19 in the United States had diabetes [1]. This proportion of decedents with diabetes is similar to the approximate proportion of older adults with diabetes, (28.8%), the group most susceptible to mortality from COVID-19 [2].

In seeking to better understand this proportion, we reviewed the references that were cited to support the report that the number of decedents from COVID-19 with diabetes was high. The first was a CDC report explaining that underlying conditions were unknown for 54.9% of COVID-19 decedents [3]. A meta-analysis of 186 observational studies, which reported that diabetes, hypertension, obesity and smoking combined contributed to nearly 30% of COVID-19 deaths, stated that the proportion of COVID-19 deaths attributable to diabetes was 8% [4]. A cohort study at 10 hospitals seeking to quantify risk of death among COVID-19 patients with prediabetes and diabetes reported that the mortality rate among those with diabetes was 27% while it was 15% among those with prediabetes [5]. The fourth reference reported similar COVID-19 mortality in those without diabetes, with prediabetes, or with diabetes that was medication-managed (by self-report) compared to those with diabetes who were not taking diabetes medication [6].

Given these discrepancies between the data cited and presentation of the data in popular media, our objective was to provide context by quantifying the proportion of decedents from COVID-19 who had pre-infection diabetes and the proportion of decedents from another respiratory virus who had pre-infection diabetes. We chose influenza as the comparator respiratory virus because of similar indications for testing and risk factors for severe infection, hospitalization, and death.

## Methods

**Fig. 1:**
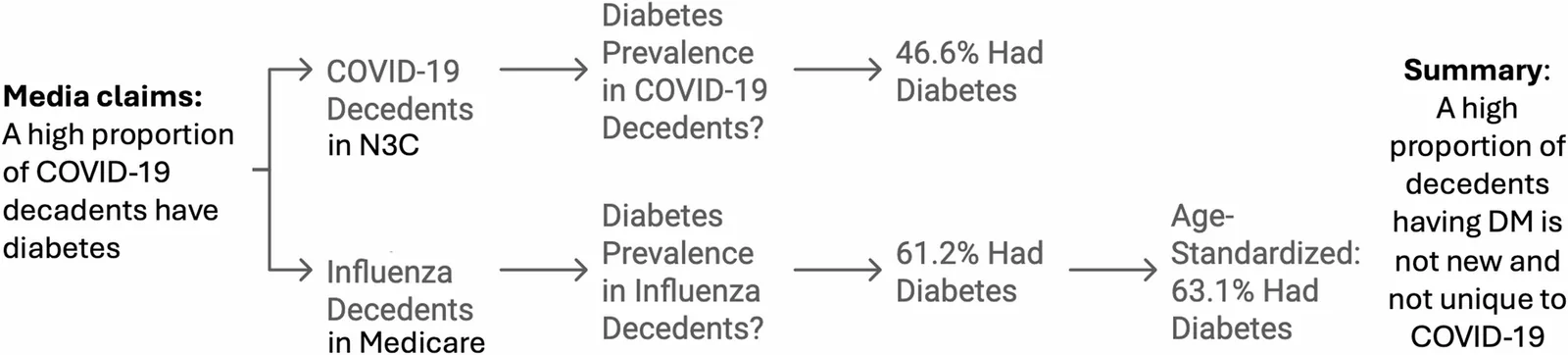
Visual summary of methods used to assess the number of influenza and COVID-19 decedents with diabetes

This analysis was approved by the University of Minnesota institutional review board (STUDY00011578), which provided a waiver of consent. We used the National COVID Cohort Collaborative (N3C) data enclave, a centralized and harmonized database of de-identified electronic health record data from 78 healthcare systems nationally. For assessing influenza decedents who had diabetes, we used Medicare data. We restricted the N3C sample to > 65 years to align with Medicare eligibility (Fig. 1).

In both databases, individuals were considered to have diabetes if they had a pre-infection diabetes. In the N3C, diabetes defined as having at least 2 outpatient (office visit or telehealth visit) diagnoses or one inpatient diagnosis for type 2 diabetes (ICD10 E11 or E13) before the index event or ever having a hemoglobin A1C (HbA1C) level > = 6.5% prior to the index date. In both databases, diabetes was defined using an international classification of disease code (ICD) 250.xx or ICD-10 E10.xx or E11.xx OR at least one prescription for an antidiabetic medication with any available look-back. In the N3C database, death due to COVID-19 was defined as inpatient mortality for the encounter in which someone was admitted for COVID-19, from March 2020 to February 2022. In the Medicare database, death due to influenza was defined using destination code from the hospital encounter in which someone was admitted for influenza, from January 2017 to December 2019. Influenza was defined by ICD-10 diagnosis code J09, J10, or J11 on the hospitalization record.

As age is an important risk factor for mortality, the age distribution of death due to COVID-19 in the N3C was calculated within 5-year age strata from 65 to 90 + years. These age-specific weights were used for direct standardization by multiplying the weights with the stratum-specific percentage with diabetes for those with in-patient mortality from influenza in Medicare. The sum of these products represents the percent of individuals dying of influenza who had diabetes, if those dying from influenza had the same age distribution as those dying from COVID-19. Two-tailed 95% confidence intervals were calculated using 0.05 as the alpha and a z-score of 1.96 as the critical value. The objective of this work was not to conduct an analysis of the risk of death associated with diabetes.

## Results

Among older adults with inpatient mortality due to COVID-19, 46.6% (95% CI: 46.1–47.0) had diabetes. Among older adults with inpatient mortality from influenza, the crude percent with diabetes was 61.2%. After age-standardizing the Medicare data to match the age distribution of the decedents in the N3C COVID-19 data, the percentage of influenza decedents with diabetes was 63.8% (95% CI: 61.5–66.1), (Table 1).

**Table 1:** Results in each database: Medicare and N3C (National Covid Cohort Collaborative)

Age (yrs)	*COVID-19*,* N3C*				*Influenza*,* Medicare*			
	No. of Deaths	No. of Decedents with DM	Percentage of Decedents with DM	Proportion in each age stratum	No. of Deaths	No. of Decedents with DM	Percentage of Decedents with DM	Weighted to the proportion in each age stratum in the N3C
65–69	8,915	4,945	55.5	0.19	206	131	63.6%	11.9
70–74	8,776	4,680	53.3	0.18	267	172	64.4%	11.9
75–79	9,249	4,490	48.6	0.19	296	201	67.9%	13.2
80–84	8,406	3,844	45.7	0.18	306	205	67.0%	11.8
85–89	6,901	2,669	38.7	0.14	327	206	63.0%	9.1
90+	5,398	1,566	29.0	0.11	316	165	52.2%	5.9
Total	47,645	22,194	46.6% (95% CI 46.1–47.0)		1718	1080	62.9%	63.8 (95% CI: 61.5–66.1)*

This table gives the number and percentage of decedents with diabetes within each 5-year age stratum (65–69 years; 70–74 years, etc.). The left most column is the 5-year age category and applies to the three Influenza, Medicare columns and the three COVID-19, N3C columns. The overall proportion of decedents with diabetes in each database is given in the Total row, with the weighted percentage in the bottom row

*Total weighted: This is the prevalence of diabetes in among older adults who experienced inpatient mortality from influenza in Medicare after weighting to the age distribution of deaths observed in N3C. This total is calculated as the sum of this product for each age stratum: 100*proportion in each age stratum in the N3C*percentage of decedents with DM in the N3C

## Discussion

This analysis leveraged N3C and Medicare data and found that among older adults with inpatient mortality from COVID-19 or influenza, a large proportion had diabetes before infection with either virus − 63% of influenza decedents had pre-infection diabetes and 47% of COVID-19 decedents had pre-infection diabetes. These findings highlight the importance of using available data to contextualize health communication to the public because a high proportion of decedents having diabetes is true for other infections and not unique to the COVID-19 virus [7].

We did not assess diabetes as an independent risk factor for mortality in patients with COVID compared to patients with influenza. Several meta-analyses have shown that diabetes is an independent risk factor for mortality from COVID-19 [8–12]. We reported only the proportion of decedents from COVID-19 who had diabetes and the proportion of decedents from influenza who had diabetes.

The large proportion of decedents, from both COVID-19 and influenza, having diabetes highlights the importance of diabetes prevention, diagnosis, and access to treatment. A meta-analysis of adults with type 2 diabetes reported two medications had high certainty of evidence for an inverse associations with COVID-19 mortality, with a summary relative risk (SRR) of 0.69 (95% CI 0.60–0.79) for metformin and 0.83 (0.71–0.97) for glucagon-like-peptide-1 receptor agonists [8]. Another study of adults with diabetes reported that metformin was associated with better survival, OR 0.65 (95% CI 0.45–0.93) [9].

Limitations: This analysis did not account for potential confounding factors including presence of other comorbid conditions like obesity, cardiovascular disease, chronic kidney disease, and socioeconomic factors in the N3C and Medicare databases [12]. This analysis should not be considered a causal analysis. Diabetes was defined via an ICD code or prescription for a diabetes medication. Using just codes or medications could lead to misclassification bias, but this bias might be expected to be similar in both databases. This analysis was limited to inpatient deaths only for both databases as the goal was to compare the proportion in each database. The potential selection bias caused by restricting to inpatient deaths may limit the generalizability of the results.

Pandemic-related strain on the healthcare system affected inpatient care during 2020–2023 compared to 2017–2019. Older adults with diabetes were negatively affected by the strain on the healthcare system, and while this was not addressed in our analysis, this strain on the healthcare system would be expected to increase the proportion of COVID-19 decedents with diabetes [13].

## Conclusion

Popular media articles implied that the proportion of COVID-19 decedents who had diabetes was elevated. This analysis demonstrates that among older adults with inpatient mortality after respiratory infection, the proportion of COVID-19 decedents who had diabetes was not more than influenza in prior years. Diabetes affects approximately 29.2% of adults aged 65 and older in the US and is a serious risk factor for mortality from viral infections [1]. These findings highlight the value of using available data to contextualize health communication to the public.

## Data Availability

The analyses described in this publication were conducted with data or tools accessed through the NCATS N3C Data Enclave https://covid.cd2h.org and N3C Attribution & Publication Policy v 1.2-2020-08-25b supported by NCATS U24 TR002306, Axle Informatics Subcontract: NCATS-P00438-B. This research was possible because of the patients whose information is included within the data and the organizations (https://ncats.nih.gov/n3c/resources/data-contribution/data-transfer-agreement-signatories) and scientists who have contributed to the on-going development of this community resource. The N3C data transfer to NCATS is performed under a Johns Hopkins University Reliance Protocol # IRB00249128 or individual site agreements with NIH. The N3C Data Enclave is managed under the authority of the NIH; information can be found at (https://ncats.nih.gov/n3c/resources). The Medicare database infrastructure used for this project was funded by the Pharmacoepidemiology Gillings Innovation Lab (PEGIL) for the Population-Based Evaluation of Drug Benefits and Harms in Older US Adults (GIL200811.0010); the Center for Pharmacoepidemiology, Department of Epidemiology, UNC Gillings School of Global Public Health; the CER Strategic Initiative of UNC’s Clinical and Translational Science Award (UL1TR002489); the Cecil G. Sheps Center for Health Services Research, UNC; and the UNC School of Medicine.
